# Environmental Exposures Relative to Locally Acquired Hansen Disease, United States

**DOI:** 10.3201/eid3107.240986

**Published:** 2025-07

**Authors:** Danielle Chaney, Jennifer Breiman, Jessica K. Fairley

**Affiliations:** Emory University Rollins School of Public Health, Atlanta, Georgia, USA (D. Chaney); Emory Healthcare, Atlanta (J. Breiman); Emory University School of Medicine, Atlanta (J.K. Fairley)

**Keywords:** Hansen disease, zoonoses, bacteria, tuberculosis and other mycobacteria, Mycobacterium leprae, environmental exposure, leprosy, armadillo, United States

## Abstract

Nine-banded armadillos (*Dasypus novemcinctus*) are suspected transmission sources of Hansen disease in North America. We conducted a telephone survey and chart review of patients with Hansen disease seen at a Georgia, USA, clinic during 1997–2022. Findings suggest frequent outdoor activities and armadillo contact were likely sources of exposure.

Hansen disease (HD), commonly known as leprosy, is caused by *Mycobacterium leprae *(*1*)*.* HD is diagnosed in approximately 150–200 persons in the United States annually, with a recent high of 225 cases in 2023 (https://www.hrsa.gov/hansens-disease), and the disease is diagnosed in 200,000 persons globally (https://www.who.int/news-room/fact-sheets/detail/leprosy). HD leads to nerve damage if left untreated, highlighting the need for early diagnosis (*1*).

Recent literature shows increasing incidence of HD in Florida (*2*), and case reports name Florida as a site of travel or residence in persons receiving an HD diagnosis elsewhere in the United States (*3*,*4*). In addition, a study in Georgia showed a recent increase in cases relating to US-born patients (*5*). The 9-banded armadillo (*Dasypus novemcinctus*), the only known animal reservoir of *M. leprae* in North America, is the suspected source of locally acquired cases (through molecular epidemiology) in the southeast and Gulf states of the United States (*6*–*8*). *M. leprae* can persist in free-living amoebae in soil, serving as another potential reservoir for human infection, which might explain the connection between human infections and the armadillo reservoir (*9*).

Recognizing a need to increase epidemiologic understanding of HD in the United States, we describe environmental exposures among patients from the southern portion of the country with suspected locally acquired HD infections. We also highlight clinical and demographic differences between cases involving US-born and immigrant persons.

## The Study

A telephone survey assessed exposures in 7 local cases involving patients who were seen at the Emory TravelWell Center (Atlanta, GA, USA). Local cases were defined as HD in patients living in the United States for at least the past 20 years without extensive travel or residence in regions with moderate to high HD endemicity. This study received approval from the Emory Institutional Review Board, and participants provided verbal consent. To provide context for the survey, we conducted a retrospective chart review of local and immigrant cases (seen at TravelWell Center during 1997–2022) (Table 1).

**Table 1:** Clinical characteristics from chart review of local and immigrant cases in study of environmental exposures in suspected locally acquired Hansen disease, United States*

Variable	Local cases, n = 13	Immigrant cases, n = 42	p value†	Odds ratio (95% CI)
Mean age at time of diagnosis, y (SD)	62 (+11)	36 (+13)	<0.005	NA
Sex			0.88	1.71 (0.36–11.24)
F	3 (23)	14 (33)	NA	NA
M	10 (77)	27 (64)	NA	NA
Unknown/not available	0 (0)	1 (2)	NA	NA
Type of disease			1.00	1.11 (0.21–5.01)
Paucibacillary	4 (31)	12 (29)	NA	NA
Multibacillary	9 (69)	30 (71)	NA	NA
Had leprosy reaction			0.032	4.23 (1.13–17.14)
Y	5 (39)	30 (71)	NA	NA
N	8 (62)	11 (26)	NA	NA
Unknown	0 (0)	1 (2)	NA	NA

*Values are no. (%) except as indicated. NA, not applicable. †2-sided p value was calculated by either *t*-test, χ^2^ test, or mid-P exact test (for an expected value <5) as appropriate, comparing immigrant cases to local cases as the reference.

**Figure 1:**
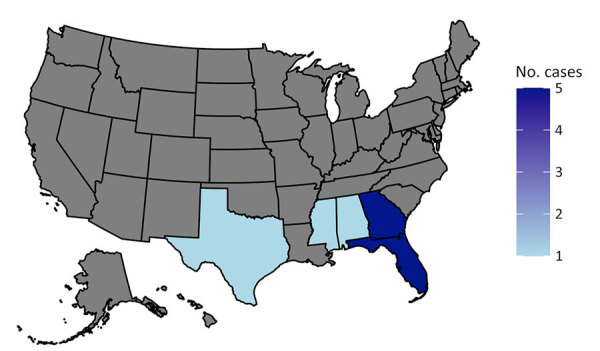
States of residence for patients in study of environmental exposures of suspected locally acquired Hansen disease, United States. A) Primary states of residence for 13 local case-patients (7 surveyed as part of study, 6 from chart review).

Seven patients participated in the telephone survey (Table 2; Appendix Table). The median age of participants was 69 years. Survey participants lived in Georgia (3 [43%]), Florida (3 [43%]), and Mississippi (1 [14%]) (Figure 1, panel A). Two Georgia participants previously resided in Florida and regularly visited the state. Five participants lived in areas with armadillos, and 5 participants had known physical contact with an armadillo or its bodily fluids. Five participants were in occupations that required them to work outdoors. One of the 2 participants who denied direct physical contact with armadillos or armadillo droppings reported gardening.

**Table 2:** Survey responses for participants in survey from study of environmental exposures in suspected locally acquired Hansen disease, United States*

**Variable**	Survey participants, n = 7
Median age, y (range)	69 (49–79)
Primary state of residence	
Georgia	3 (43)
Florida	3 (43)
Mississippi	1 (14)
Lived internationally	
Y†	3 (43)
N	4 (57)
International travel in the past 10 y	
Y‡	4 (57)
N	3 (43)
Live in areas with armadillos	
Y	5 (71)
N	2 (29)
Physical contact with armadillos	
Y	3 (43)
N	4 (57)
Contact with armadillo bodily fluids or droppings	
Y	3 (43)
N	4 (57)
Time spent outdoors per week, h	
0–2	0 (0)
3–5	4 (57)
6–8	0
≥9	3 (43)
Occupation requires working outside	
Y	5 (71)
N	2 (29)
Takes part in at least one outdoor activity	
Y	6 (86)
N	1 (14)
Outdoor activities reported	
Gardening	4 (57)
Hiking	2 (29)
Hunting	2 (29)
Camping	3 (43)
Outdoor swimming	1 (14)
Other activity§	5 (71)

*Values are no. (%) except as indicated. †Participants reported living in Germany, Morocco, and Canada. ‡Participants reported travel to The Bahamas, Australia, Spain, France, Germany, Belgium, England, Scotland, Estonia, Denmark, Finland, Norway, Russia, Japan, Mexico, and Canada. §Other outdoor activities reported included kayaking, golfing, archery, and fishing.

**Figure 2:**
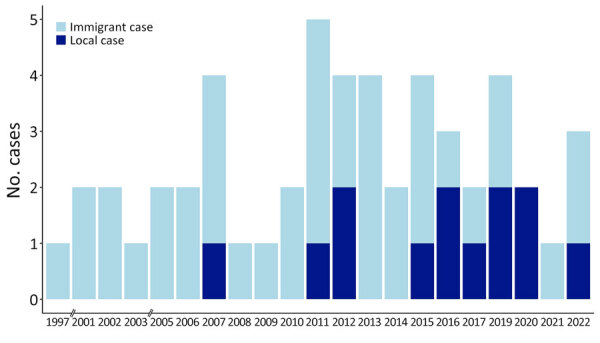
Number of Hansen disease cases from chart review in study of environmental exposures of suspected locally acquired Hansen disease, by year of diagnosis, United States. A total of 55 cases were included in the review.

We included 55 patients in the chart review (13 presumed local, including the 7 survey participants, and 42 immigrant), noting the year of diagnosis for each (Figure 2). Most immigrant cases originated from Brazil (11 [26%]), Mexico (6 [14%]), or India (6 [14%]) (Figure 1, panel B). The mean age at time of diagnosis was 62 (SD +11) years for local cases and 36 (SD +13) years for immigrant cases (p<0.005). Patients with leprosy reactions were more likely to be from outside the United States (Table 1). None of the local cases had a known content with HD.

On the basis of the survey responses, living in an environment with armadillos and spending time outdoors were very frequently reported, lending possible exposure routes to *M. leprae* (*6*–*8*). Most interviewees reported direct physical contact with an armadillo or its bodily fluids in their lifetime, and >70% lived in an area where they see armadillos. We posited that persons who did not report physical contact acquired the bacterium through indirect means, considering that multiple studies have confirmed potentially viable *M. leprae* in environmental sources (*10*–*12*). In fact, most interviewees regularly took part in outdoor activities, such as golfing and gardening, where they might have come in contact with soil or grass contaminated with armadillo urine or feces, which might have served as the route of transmission. In addition, >70% of interviewees worked in outdoor occupations, a much higher percentage than the 2020 national average of 4.3% (*13*). Given the high level of reported outdoor activity of survey participants, we considered also that transmission of HD might have occurred via other zoonotic reservoirs, such as ticks, which have demonstrated the ability to harbor *M. leprae* (*14*).

## Conclusions

The participants in this survey lacked typical risk factors for acquiring HD; none lived in or spent significant time in countries with high endemic HD or lived with or knew anyone with HD. We speculated that transmission, thus, must have occurred within the United States. With studies linking the 9-banded armadillo to locally acquired cases in the southern United States (*6*,*7*), combined with the similar exposure profiles of our survey participants to both armadillos and soil where armadillos live, we believe data support armadillos as a possible route of transmission. Literature is scarce regarding risk factors for HD in the United States, and anecdotal evidence from HD experts across the United States suggests that cases in US-born persons are rare outside of southern states. A recent study of presumed locally acquired HD cases in California identified 6 patients lacking traditional risk factors, such as geographic location or contact with an infected person. Although only 1 patient in that cohort reported armadillo exposure, occurring >50 years prior to diagnosis, all of the patients reported travel to either an endemic country or the US Gulf Coast (*4*).

We did find a statistically significant difference in age of diagnosis between local and immigrant cases; local case-patients were older. In addition, persons with leprosy reactions had higher odds of being from another country. We considered the possibility that US-born patients have different exposure risks to HD compared with patients born outside the United States. For instance, persons from countries with a higher HD prevalence might have a higher likelihood of encountering the bacteria and becoming infected at an earlier age. For person-to-person transmission to occur, research suggests that prolonged close contact with an untreated person is needed (*15*). If zoonotic transmission—as opposed to person-to-person transmission—is the primary source of infection in the United States, and given that *M. leprae* infection was not detected in southeastern US armadillo populations before 2009 (*6*), exposure opportunities for US-born persons compared with persons from endemic countries would be notably different. A counterpoint to this theory is that *M. leprae* is slow-growing, and disease manifestation can take years (*15*). In addition, children and young adults in the United States typically are heavily exposed to dirt and the outdoors, and thus hypothetically exposed to HD, making it highly possible that local case-patients are actually becoming infected earlier than data indicates, but clinical manifestations are occurring at a later age.

The primary limitation of this study was the small sample size, limiting the generalizability of the findings. For our results to be more widely applicable, a demographically and geographically diverse patient population would need to be sampled, an undertaking that would be difficult with this rare disease. Another limitation is the lack of routine molecular genotyping of *M. leprae* isolates from US patients. Comparing patient strains to those associated with armadillos or other reservoirs could clarify sources of infection and inform public health and clinical messaging.

Our study demonstrates the importance of studying HD in a low-endemic setting because further research into the transmission and host-pathogen interactions in the United States could shed light on the larger-scale questions plaguing HD researchers worldwide, namely, how the bacteria is transmitted and who is most at risk for disease. Early diagnosis and treatment of cases of HD in the United States depends greatly on a clear understanding of the risk factors for transmission.

Appendix Telephone survey questions and response options from environmental exposures of suspected locally acquired Hansen’s disease, USA
