# Supplementary material for: Environmental Exposures Relative to Locally Acquired Hansen Disease, United States
Source: Emerg Infect Dis. 2025 Jul;31(7):1437–41. doi: 10.3201/eid3107.240986 (PMC12205374; doi:10.3201/eid3107.240986)
Supplement: Appendix — Telephone survey questions and response options from environmental exposures of suspected locally acquired Hansen’s disease, USA [file 24-0986-Techapp-s1.pdf]

Article DOI: http://doi.org/10.3201/eid3107.240986

EID cannot ensure accessibility for supplementary materials supplied by authors. Readers who have difficulty accessing supplementary content should contact the authors for assistance.

Environmental Exposures Relative to Locally Acquired Hansen Disease, United States

Appendix

Appendix Table: Telephone survey questions and response options from environmental exposures of suspected locally acquired Hansen's disease, USA.

Code	Question	Response Options
**Section A: Demographics, Residential, and Travel History**		
A1	How old are you?	Open-ended response
A2	What state do you primarily reside in?	Open-ended response
A3	How many years have you lived in that state?	Open-ended response
A4	What type of environment do you primarily live in?	Rural; Suburban; Urban
A5	How many years have you lived in that environment?	Open-ended response
A6	Have you ever lived outside of the United States?	Yes/No
A7	What country/countries did you live in?	Open-ended response
A8	How many years did you live in each country?	Open-ended response
A9	Have you traveled outside the United States in the past 10 y?	Yes/No
A10	What countries have you traveled to?	Open-ended response
**Section B: Work and Recreational Exposures**		
B1	What do you do for work?	Open-ended response
B2	Does your occupation require you to work outside?	Yes/No
B3	About how many hours per week do you spend outside?	0–2 h; 3–5 h; 6–8 h; 9+ h
B4	Which of the following outdoor activities, if any, do you take part in?	Gardening; Hiking; Hunting; Camping; Outdoor swimming; None of the above
B5	Do you regularly perform any other non-work-related outdoor activities not listed above?	Yes/No
B6	Which activities do you perform?	Open-ended response
B7	Do you frequently have direct contact with soil?	Yes/No
**Section C: Rodent and Armadillo Exposure**		
C1	Do you live in an area where you see armadillos?	Yes/No/Do not know
C2	Are armadillos pests around your home?	Yes/No/Do not know
C3	Have you ever had physical contact with an armadillo such as hunting, eating, touching, or keeping an armadillo as a pet?	Yes/No/Do not know
C4	What kind of physical contact did you have?	Hunting; Eating; Touching; Keep as pet; Other (specify)
C5	Have you ever had physical contact with armadillo droppings or bodily fluids?	Yes/No/Do not know
C6	Have you ever noticed armadillo droppings or bodily fluids near your home?	Yes/No/Do not know
C7	Do you believe there has been an increase in armadillos around your home in the past 5 y?	Yes/No/Do not know
C8	Do you remember seeing armadillos around your home as a child?	Yes/No/Do not know
C9	Do you remember having physical contact with an armadillo as a child?	Yes/No/Do not know
C10	Have you ever had physical contact with a rabbit such as hunting, eating, touching, or keeping a rabbit as a pet?	Yes/No/Do not know
C11	What kind of physical contact did you have?	Hunting; Eating; Touching; Keep as pet; Other (specify)
C12	Have you ever had physical contact such as hunting, eating, touching, or keeping as a pet with any other small animals like squirrels, mice, or rats?	Yes/No/Do not know
C13	What kind of physical contact did you have?	Hunting; Eating; Touching; Keep as pet; Other (specify)
**Section D: Miscellaneous Questions**		
D1	Have you ever been told by a doctor that you have a Vitamin D deficiency?	Yes/No/Do not know
D2	How do you think you got Hansen's Disease, if you have any ideas about that?	Open-ended response
